# Metabolic and molecular mechanisms underlying the foliar Zn application induced increase of 2-acetyl-1-pyrroline conferring the ‘taro-like’ aroma in pumpkin leaves

**DOI:** 10.3389/fpls.2023.1127032

**Published:** 2023-01-26

**Authors:** Liting Deng, Xian Yang, Yuehan Qiu, Jianning Luo, Haibin Wu, Xiaoxi Liu, Gangjun Zhao, Hao Gong, Xiaoming Zheng, Junxing Li

**Affiliations:** ^1^ Guangdong Key Laboratory for New Technology Research of Vegetables, Vegetable Research Institute, Guangdong Academy of Agricultural Sciences, Guangzhou, Guangdong, China; ^2^ College of Horticulture, South China Agricultural University, Guangzhou, Guangdong, China

**Keywords:** 2-acetyl-1-pyrroline, aroma, pumpkin, metabolome, transcriptome, Zn

## Abstract

**Introduction:**

Fresh pumpkin leaf is popular vegetable for its rich nutrition. The pleasant taro-like odour is important aroma quality of crops, and mostly contributed by 2-acetyl-1-pyrroline in pumpkin. Element Zn can impact metabolite biosynthesis in plants, including aroma formation. However, Zn-induced biochemical responses, especially 2-acetyl-1-pyrroline formation in pumpkin, haven’t been elucidated.

**Methods:**

This study integrated metabolome and transcriptome to explore molecular fluctuations in pumpkin leaves at different time intervals after foliar Zn treatment.

**Result and Discussion:**

We first identified more than one thousand metabolites from pumpkin leaves by integrating different mass spectrometry methods according to the form in which a metabolite exists. Comparative metabolomic analysis revealed there were separately 25 out of 50 and 286 out of 963 metabolites that were respectively identified by gas chromatography-mass spectrometry and liquid chromatography-tandem mass spectrometry, differentially regulated by Zn treatment. Our findings revealed that 50mg/L of Zn significantly enhanced 2-acetyl-1-pyrroline production by more than 38%, which was contributed by increased biosynthesis of its precursors, including ornithine and proline. The following transcriptome analysis discovered 30,574 genes, including 953 novel genes. Zn treatment induced the differential expression of 41.6% of identified genes which were supposed to regulate the downstream metabolite changes in a time-dependent manner. Pathway analysis indicated that alternations in primary metabolism, including carbon metabolism and biosynthesis of amino acids, were vital to the fluctuated aromatic compound generation. Phytohormones and transcription factors may regulate the expression of gene *P5CS* and proline biosynthesis, which, therefore, affect 2-acetyl-1-pyrroline production. This research reveals molecular mechanisms of 2-acetyl-1-pyrroline formation in pumpkin, which will provide the molecular basis for desired aroma compound production through metabolite engineering.

## 1 Introduction

Aroma compounds are one of the biggest classes of metabolites, and important factors that impact the character and quality of food, and economic value, including vegetables, fruits, and beverages (Zeng et al., 2019). Only a few key odorants with significant concentration and exceeding their odour thresholds can predominantly contribute to the overall aroma profiles of plants, and therefore be detected by the human olfactory system (Poehlmann and Schieberle, 2013). Therefore, the characteristic aroma component, especially those that act as the principal contributors to the pleasant odour, becomes the vital agronomic and economic trait of plants. Cucurbitaceae plants are the species that provide most food for human consumption, among which pumpkin is a well-known source for the accumulation of different metabolites and valuable nutrients (Mashiane et al., 2021). Except for fruits, pumpkin leaves are believed to be safe and nutritious vegetables, and with great popularity in diets of Asian and African consumers (Mashiane et al., 2021). Consumer liking is strongly correlated with aromatic compounds. Pumpkin leaves generate various metabolites, and produce rich aroma. On the market, pumpkin varieties with distinctive taro-like flavour are more preferred. The successful and accurate distinguishment of different pumpkin varieties with and without taro-like odour sensing from either leaves or fruits by the characteristic component 2-AP proved it is the key contributor of the distinctive odour (Li et al., 2019; Li et al., 2022). However, mechanisms related to 2-AP production and accumulation in pumpkin are not clearly covered. Additionally, the involvement of both internal genetic factors and external environmental changes in the regulation of 2-AP formation increases the complexcity of associated mechanisms.

In nature, metabolites generation is a biochemical and physiological response to environmental stresses that plants derived during the evolutionary process. It is interesting that these generated metabolites from stress responses can be harmful to plants or potentially improve the important quality components in crops (Zeng et al., 2019). For instance, stress application to leaves impacts the aroma biosynthesis and tea quality (Zeng et al., 2019). Hence, artificially applying safe and effective stimulates is of great importance to enhance the production of the characteristic compounds and improve aroma quality of vegetables. In practice, exogenous application of micronutrients at low concentration can change the aroma formation, therefore improving the aromatic characteristics of plants (Bao et al., 2021). For instance, increased number of volatile metabolites and their total concentrations were observed in grape after foliar Zinc sulfate (ZnSO_4_) treatment (Song et al., 2016). Zinc chloride (ZnCl_2_) application positively impacted the yield, together with the aroma of fragrant rice, especially the accumulation of key aroma contributor 2-AP (Bao et al., 2021). It is well-known that Zn is an essential microelement in plants, and involves in the regulation of diverse physiological and biochemical processes at all developmental and metabolic stages (Broadley et al., 2007). It is reported that Zn-mediated changes in carbohydrate metabolism and biosynthesis of plant hormones were involved in the regulation of 2-AP production in fragrant rice (Bao et al., 2021). Potentially, exogenous application of Zn could impact the aroma production in pumpkin. However, the effect of Zn element on the 2-AP production in pumpkin, and the underlying regulatory mechanisms has not been demonstrated yet.

It is suggested that 2-AP could be initially synthesised in leaves of the fragrant rice (Hinge et al., 2016; Bao et al., 2021). Foliar Zn application could be a direct and operable cultivation technique for enhancing the 2-AP content and improving the taro-like flavour of pumpkin leaves during the process of agricultural production. Revealing the molecular mechanism underlying the Zn-induced changes of aroma formation and increase of 2-AP content is vital to help a more accurate and effective application of exogenous elements in improving the aroma quality of pumpkin leaves, as well as the new aromatic pumpkin cultivars through genetic engineering.

Omics are imperative in discovering how plants undergo complex biological processes as associated techniques are effective in the rapidly expanding collection of biomolecule information in plants. In this study, metabolome and transcriptome analysis were applied to pumpkin leaves at different time-points after foliar Zn treatment. The omic data was interpreted and integrated to obtain a more comprehensive understanding of involved biomolecules and their interrelationships, and their roles in 2-AP accumulation and aroma formation. The research found that Zn significantly enhanced the 2-AP concentration in pumpkin leaves. It was manifested that the Zn-induced responses in pumpkin leaves were at both metabolite level and gene level. The improved biosynthesis of 2-AP precursors, such as ornithine and proline, and associated genes contributed to the accumulation of 2-AP. Hormones and transcription factors (TFs) were involved in the regulation of 2-AP biosynthesis. Our research elucidated that Zn application could be an effective technique to improve the aromatic profile of the taro-like flavours of pumpkin leaves. The illustrated molecular mechanisms underlying Zn-induced increase of 2-AP will be of great value to the quality breeding of pumpkins associated with aroma formation.

## 2 Materials and methods

### 2.1 Plant materials

The pumpkin variety NO.44, belonging to the *Cucurbita moschata* D., was selected as the experimental material. This material is the selfed progeny of one important pumpkin germplasm that has the taro-like aroma and is widely used for pumpkin fragrance-associated breeding in China. Seeds were germinated at 30°C in the dark for two days and then cultured in Hoagland’s hydroponic medium which contains all essential elements at proper levels for growth. Treatment was carefully applied to one-month-old pumpkin through evenly spraying ZnCl_2_ (60 mL, 50 mg/L) on each leaf surface according to the previous experiments (Bao et al., 2021; Imran et al., 2022). The treatment and concentration of ZnCl_2_ was determined according to previous studies as the specified application significantly improved the 2-AP abundance of pumpkin (not published). After foliar Zn application, fresh pumpkin leaves at top three nodes were separately collected at 24 hrs, 72 hrs, and 120 hrs. Plant samples sprayed with MilliQ water were taken as the controls. Three biological replicates were prepared for each condition, and three plants were collected for each replicate. The three experimental conditions (Zn_24 hrs, Zn_72 hrs, and Zn_120 hrs), together with the control (Ctrl), constituted the experimental materials in this study. Collected tissues were snap-frozen in liquid nitrogen tank, followed by storage inside an ultra-low temperature freezer at -80°C for the subsequent experiments.

### 2.2 Volatile metabolites analysis using GC-MS

Volatile compounds were analyzed *via* the headspace solid-phase microextraction (HS-SPME) combined with GC-MS analytical technology. Samples were freeze-dried in a vacuum and ground into powder. Half gram of lyophilized and powdered leave sample was weighed and carefully placed in a 20 mL vial. 3-nonanone and 2,4,6-trimethylpyridine (2 μL, 50μg/mL) were added to the vial as the dual internal standards (Fan et al., 2021; Lan et al., 2021; Li et al., 2022; Lu et al., 2022). The vial was sealed immediately and equilibrated at 70°C for five minutes. DVB/CarbonWR/PDMS Smart SPME Arrow (1.1 mm, 120/20 μm) (CTC Analytics AG) was used for the extraction of volatile metabolites. After 30 min of extraction and absorption at 70°C, SPME was desorpted and sample was analyzed on 8890-5977B GC/MS (Agilent Technologies, USA). The GC temperature program started at 35°C (2 min), then rose up to 100°C (5 min) at a rate of 5°C/min, and finally kept at 215 °C. The mass spectrometer was operated in electron impact (EI) mode with the ionization energy of 70 eV. The ion source temperature was programmed as 230°C. The full scan mode was applied with a mass scan range of 35-450 amu. The relative abundance of the compound was determined with a method related the peak areas of volatiles to that of the internal standard. The downstream comparative analysis was applied to the identified metabolites. A metabolite was considered with changed abundance if the result of statistical analysis was significant (p-value lower than 0.05).

### 2.3 Nonvolatile metabolites analysis using LC-MS/MS

The metabolomic profiling experiments were conducted by Metware, Wuhan. The pumpkin leave samples were processed in a vacuum freeze dryer (Scientz-100F). The freeze-dried leaves were then grounded (MM 400, Retsch). 100 mg of leave power was weighed and used for metabolite extraction. Metabolites were extracted using 70% cold methanol. Samples were vortexed for 30 sec every 30 min, after six times of vortex, the samples were kept in the extraction solution at 4°C for overnight. High speed centrifuge (10,000 g, 10 min) was followed after the overnight extraction. The supernatant was carefully transferred onto the filter membrane (0.22 μm pore size). The filtered sample was kept in vial before loading to the instrument for mass spectrometer analysis (MS, 4500 Q TRAP, Applied Biosystems, MA, United States) coupled to an ultra performance liquid chromatography (UPLC, Shim-pack UFLC SHIMADZU CBM30A system, Kyoto, Japan). Metabolites were characterized using the multiple reaction monitoring method, and then searched against the MetWare database. The downstream data analysis was carried out using Analyst 1.6.1 software. Metabolites were considered with changed abundance if the result of statistical analysis was significant (p-value lower than 0.05).

### 2.4 Transcriptome analysis

RNA extraction was performed using the Trizol reagent supplied by TaKaRa, Japan, according to the instructions. The RNA integrity and purity were examined through the agarose gel electrophoresis experiment. Qubit 2.0 fluorometer (Thermo Fisher Scientific, MA, USA) was used to measure the RNA concentration. The RNA quality was further confirmed using the Agilent 2100 Bioanalyzer (Agilent, CA, United States) followed by cDNA library construction. The constructed cDNA library was validated through quality control assays. Library concentration and size distribution were confirmed through Qubit dsDNA high sensitivity assay and Agilent ScreenTape assay, respectively. Kapa BioSystems qPCR method was applied to calculate molarities and normalize libraries prior to pooling and sequencing. The sequencing data was uploaded to the CNGB Sequence Archive (CNSA) of China National Genebank DataBase (CNGBdb) (accession number CNP0003866). Clean reads were aligned to the *Cucurbita moschata* reference genome (http://cucurbitgenomics.org/). Differentially expressed genes were analysed using DESeq2 package. The statistical significance testing approach was applied to identify genes that express differently (p-value less than 0.05) (Conesa et al., 2016). The functions of identified genes were annotated using the GO, KEGG and Nr databases.

### 2.5 Quantitative real-time PCR

The RNA and cDNA samples were well-prepared, and experiment was conducted as previously described (Deng et al., 2016). Actin was selected as the control for the normalization of gene expression levels in pumpkin leaves (Liu et al., 2021). Primers for gene amplification (**Table S8**) were designed using Primer Premier 5. Experiment was conducted with the SYBR Premix Ex Taq Kit (TaKaRa, Kusatsu-Shiga, Japan) according to the protocol. The RT-qPCR reaction mixture system consisted of 1 μL cDNA, 5 μL SYBR Green PCR Master Mix (Takara), 0.3 μL of 10 μM forward primer, 0.3 μL of 10 μM reverse primer, and 3.4 μL ddH2O to a final volume 10 μL. qRT-PCR was performed on the CFX96 machine (Bio-Rad, CA, United States) under the following condition: 30 s at 95°C followed by 40 cycles (5 s at 95°C, 30 s at 60°C) and melting curve analysis at 65-95 °C. Relative expression levels of targeted genes were determined with the 2^-ΔΔCT^ method (Deng et al., 2016). The expression level of each gene was calculated as the average of that of all three replicates from different condtions.

## 3 Results

### 3.1 Metabolite changes in pumpkin after foliar zn application

2-AP is highly volatile. According to the form that a metabolite exists, GC-MS was applied to detect the impact of Zn application on the formation of 2-AP and other aroma volatiles in pumpkin leaves. As expected, 2-AP was detected by GC-MS, and had significantly increased abundance at 24 hrs and 72 hrs. Including 2-AP, there were 50 volatile compounds identified from volatile emissions (**Table S1**), among which 25 metabolites changed their abundance after various Zn treatments. In addition, geraniol, which is an important terpene alcohol and gives some aromatic plants the rose-like pleasant fragrance, was also detected through the GC-MS approach, and increased abundance was found at 24 hrs. However, 3-hexen-1-ol, which is well-known for its strong freshly cut grass-like aroma, was decreased at all stages after Zn application. The changed abundance of different aromatic compounds determines the overall aroma profile of pumpkin.

**Figure 1:**
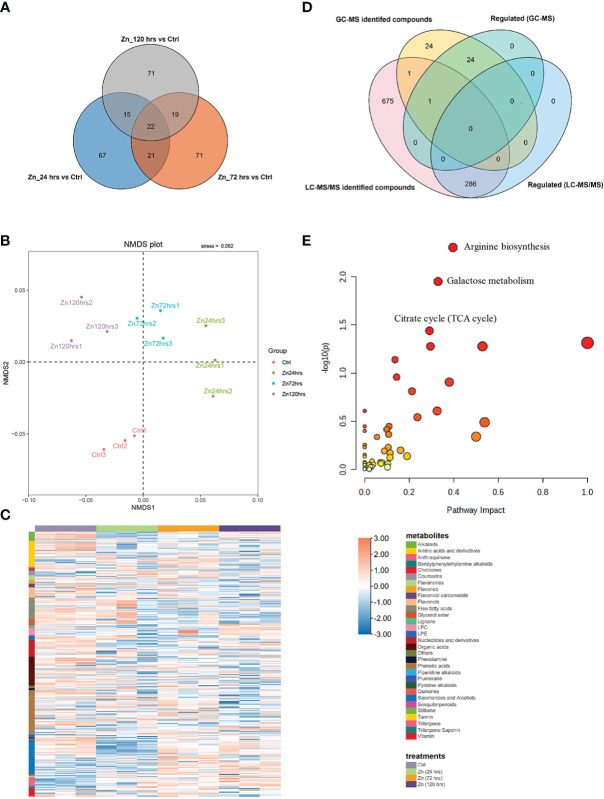
Metabolite changes in pumpkin induced by Zn application. **(A)** Venn diagram indicating the overlaps between the differentially expressed nonvolatile metabolites in the pumpkin at 24 hrs, 72 hrs, and 120 hrs after Zn treatment, respectively (p value < 0.05). **(B)** NMDS (non-metric multidimensional scaling) analysis represents the rank orders of four experimental conditions after clustering (the stress value was 0.052, which was lower than 0.1). **(C)** Heatmap generated with the Zn regulated metabolites or substances in the pumpkin. Orange and blue colors separately indicate the relative high or low abundance of metabolites in four different experimental conditions. The column and row banners represent the classifications of different treatments and component classes, respectively. **(D)** Venn diagram indicating the overlaps between the GC-MS identified volatiles and LC-MS/MS identified nonvolatiles in the pumpkin at 24 hrs, 72 hrs, and 120 hrs after Zn treatment, respectively. **(E)** The scatter plot shows enriched and matched pathways using all regulated compounds that were successfully annotated using the database in MetaboAnalyst (only the top three enriched pathways were labelled). The x and y axes are pathway impact values from the pathway topology analysis, and the corresponding p value from the pathway enrichment analysis, respectively.

Followingly, liquid chromatography coupled with tandem mass spectrometry (LC-MS/MS) was utilized to detect metabolites in the pumpkin leaves at different time intervals after foliar Zn treatments, with the aim to integrate with the GC-MS generated data and explore the intermediates or precursors associated with the identified 2-AP and its biosynthesis. By contrast, more abundant metabolites (963 metabolites) were identified from pumpkin leave using LC-MS/MS (**Table S2**). Zn-induced fluctuations in metabolite generation were excavated through the downstream bioinformatic analysis. There were 286 metabolites changed in their abundance, which account for 29.7% of the identifications by LC-MS/MS (**Figure 1A**). Detailly, there were separately 125, 133, and 127 metabolites regulated in the pumpkin at 24 hrs, 72 hrs, and 120 hrs after the foliar application of Zn. The classification analysis through the non-metric multidimensional scaling (NMDS) method found different experimental conditions (Zn24hrs, Zn72hrs, Zn120hrs, and Ctrl) were well-clustered and separated, indicating Zn treatments affected the metabolite production (**Figure 1B**). The regulated metabolites were analyzed and categorized into specific groups (**Figure 1C**; **Table S2**). Phenolic acid, saccharide and alcohols, organic acids, amino acids and derivates, and free fatty acids were proved to be the largest five categories, with 47, 37, 31, 28, and 23 compounds being regulated by Zn treatment. Comparative analysis indicated that variated production of those commpounds after Zn treatment. For instance, most free fatty acids were decreased (19 out of 23 compounds), especially at 72 hrs and 120 hrs after Zn application. However, the ten regulated triterpene metabolites were all increased at different time-points after Zn treatment. There were four, four and three Zn-regulated triterpene compounds at 24 hrs, 72 hrs, and 120 hrs, respectively, with fold change ranged from 1.62 to 6.55.

Venn analysis (**Figure 1D**) between volatile and nonvolatile metabolites separately identified using GC-MS and LC-MS/MS indicated that there were few overlaps of identifications except for 1-decanol and benzaldehyde, demonstrating the importance of integrating different mass spectrometry methods for illustrating complex metabolic changes. Importantly, the metabolite analysis which ultilized and integrated different MS methods identified 2-AP, and 2-AP formation closely related compounds, including pyrroline, ornithine, and proline. Increased concentrations of 2-AP, ornithine, and proline were specially recognized at 24 hrs and (or) 72 hrs compared to the control, indicating the 2-AP generation and accumulation could be related to the biosynthesis and metabolism of amino acids, as the previous study reported. Compounds of which abundance were regulated by Zn treaments were further annotated, followed by pathway analysis using MetaboAnalyst (https://www.metaboanalyst.ca/). Arginine biosynthesis, galactose metabolism, and citrate cycle were the top three significantly enriched pathways (**Figure 1E**), and there were separately six, seven, and five compounds with regulation classified into the three pathways according to their orders mentioned above.

### 3.2 Gene expression dynamics in pumpkin induced by Zn

**Figure 2:**
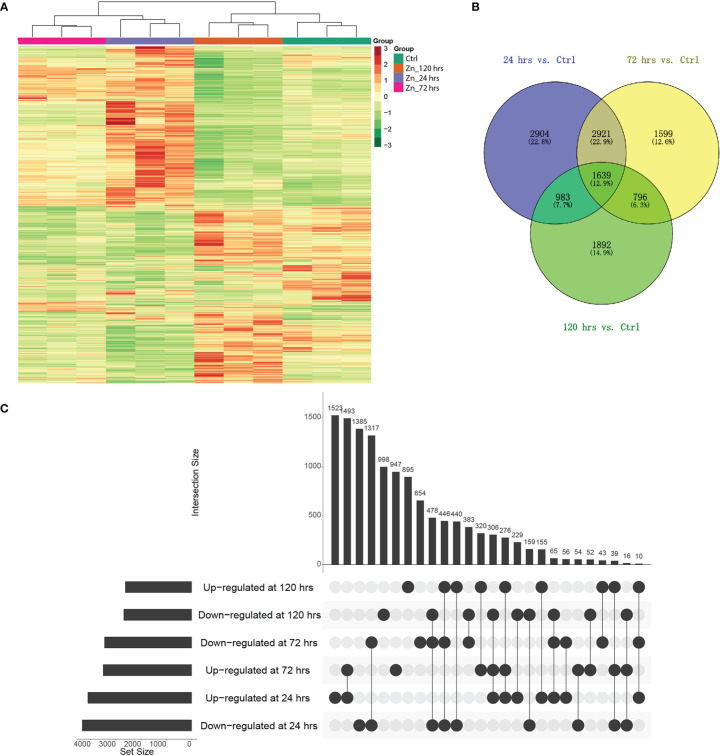
Differential gene expression in pumpkin at different time-points after Zn induction. **(A)** Heatmaps generated with differentially expressed genes after Zn treatments. Column colors indicate treatment type. Red and green colors indicate the relatively higher or lower expression levels of genes, respectively. **(B)** Venn diagram indicating the overlaps of differentially expressed genes in the pumpkin at three time points (p_value < 0.05). **(C)** UpSet visualisation of the number of regulated genes at different conditions compared to the control. Filled circles and vertical lines represent the corresponding genes being compared. The left bar graph for the UpSet plot shows the number of significantly increased or decreased gene changes in each of six comparisons upon Zn treatment.

The expression of genes was investigated to explore the potential molecular mechanisms underlying the Zn induced metabolite changes, especially the regulation associated with the 2-AP production. Transcriptome analysis discovered 30,574 genes, including 953 novel genes (**Table S3**). Comparative analysis found 41.6% of identified genes in pumpkin leaves responded to Zn treatment. A total of 12,734 genes significantly changed their expression levels, and the most regulated genes were found at 24 hrs which was the earliest stage after the Zn application in this study (**Figures 2A, B**). At different time points, there were separately 8447 (4,113 increased; 4,334 decreased), 6,955 (3,503 increased; 3,452 decreased), and 5,310 (2,624 increased; 2,686 decreased) genes with changed abundance at 24 hrs (**Table S4**), 72 hrs (**Table S5**), and 120 hrs (**Table S6**). Additionally, the regulation of genes was impacted by Zn in a time-dependent manner. Similar yet different regulations of gene expression were discovered among distinct treatments when compared to the control condition (**Figure 2C**). Genes with the same regulation patterns (either increased or decreased abundance) were mostly identified between 24 hrs and 72 hrs.

### 3.3 Integration analysis of metabolome and transcriptome data

**Figure 3:**
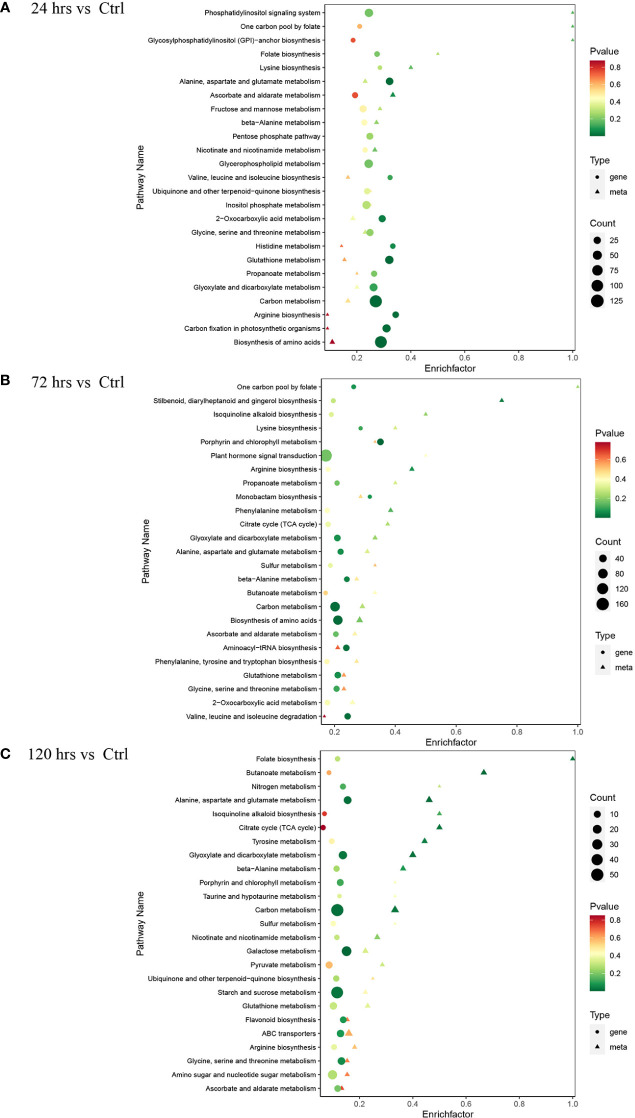
KEGG pathway enrichment analysis of differentially expressed genes and metabolites in the pumpkin at different time-points (only the top 25 significantly enriched pathways were displayed). **(A)** 24 hrs. **(B)** 72 hrs. **(C)** 120 hrs. The advanced bubble chart shows the enrichment of differentially expressed genes in various pathways. The y-axis represents pathways, and x-axis represents the enrich factors (enrich factor refers to the ratio of the number of regulated genes enriched in the pathway and the total amount of genes in the background in the same pathway). The greater rich factor indicates a higher enrichment. The size and colours (green, yellow, and red) of the bubble separately represent the amount of differentially expressed genes enriched in the pathway and its enrichment significance. The bigger bubble represents more associated genes regulated and enriched in the corresponding pathway. The smaller p-value represents better enrichment.

The metabolome and transcriptome data were integrated to illustrate the molecular changes on both metabolite and gene levels in pumpkin after Zn treatments. The enrichment analysis identified various pathways with regulations in metabolites and associated genes (**Figure 3**). It is evident that regulated genes were significantly enriched in pathway carbon metabolism after Zn treatments (24, 72, and 120 hrs) when compared to the normal condition. More genes with expression changes were found in pathway biosynthesis of amino acids at both 24 hrs (**Figure 3A**) and 72 hrs (**Figure 3B**). Responded genes involved in plant hormone signal transduction were especially increased in pumpkin after the Zn treatment for a relatively longer time-point (72 hrs). More remarkable fluctuations in pathway starch and sucrose metabolism and associated genes were observed at 120 hrs time-point after Zn application (**Figure 3C**).

**Figure 4:**
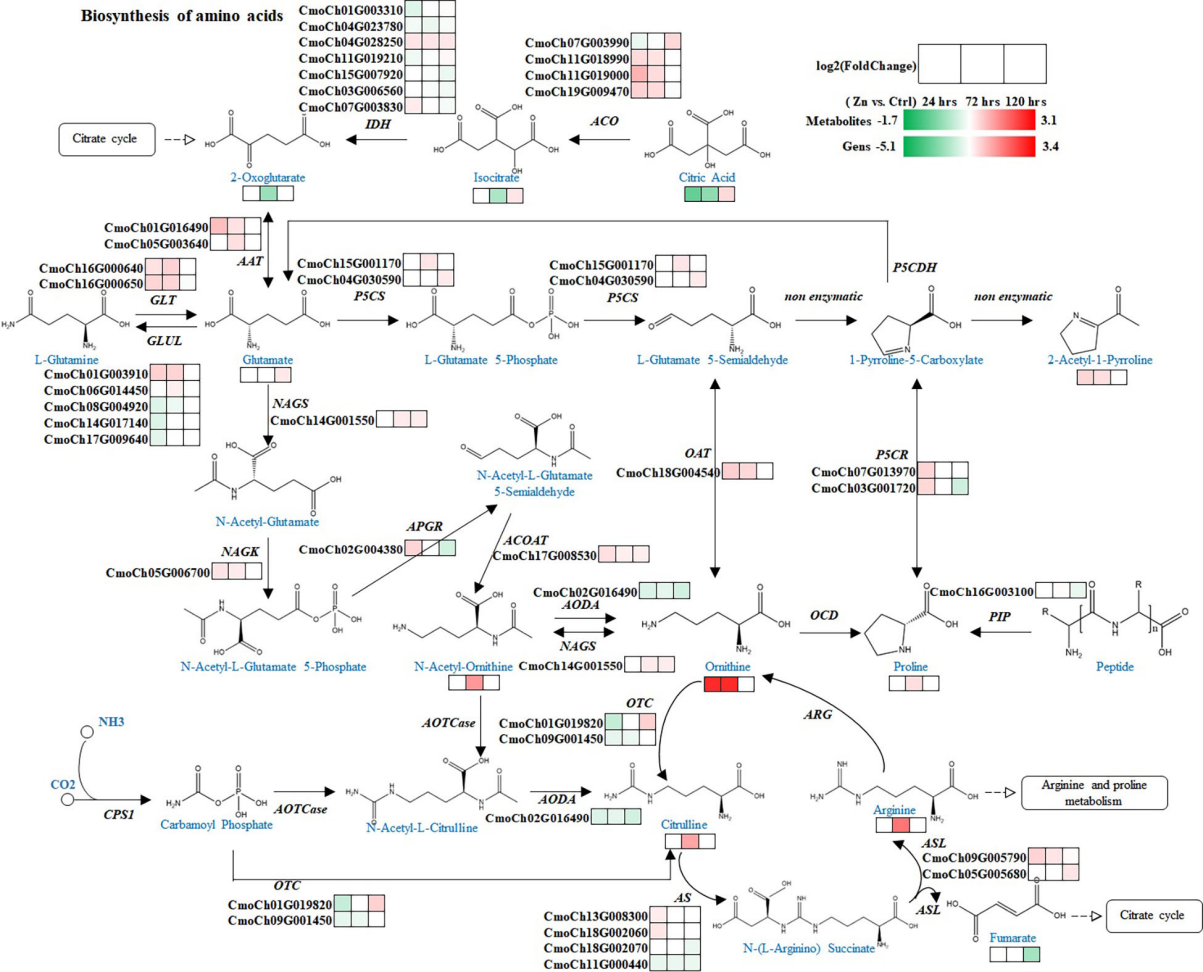
Regulation of pathways related to 2-AP biosynthesis. Blue and black colors are metabolites and genes involved in the pathway, respectively. Heatmaps illustrate the regulations of metabolites and genes at different time-points after Zn treatment. Red and green colors represent increase and decrease in abundance of metabolites or genes. Abbreviations, full names and EC numbers of enzymes: *ACO* aconitate hydratase [EC:4.2.1.3]; *IDH* isocitrate dehydrogenase [EC:1.1.1.42]; *AAT* aspartate aminotransferase [EC:2.6.1.1]; *GLT* glutamate synthase [EC:1.4.1.14]; *GLUL* glutamine synthetase [EC:6.3.1.2]; *P5CS* delta-1-pyrroline-5-carboxylate synthetase [EC:2.7.2.11, 1.2.1.41]; *NAGS* glutamate N-acetyltransferase [EC:2.3.1.35, 2.3.1.1]; *NAGK* acetylglutamate kinase [EC:2.7.2.8]; *APGR* N-acetyl-gamma-glutamyl-phosphate reductase [EC:1.2.1.38]; *ACOAT* acetylornithine aminotransferase [EC:2.6.1.11]; *AODA* acetylornithine deacetylase [EC:3.5.1.16]; *OCD* ornithine cyclodeaminase [EC:4.3.1.12]; *PIP* proline iminopeptidase [EC:3.4.11.5]; OAT ornithine–oxo-acid transaminase [EC:2.6.1.13]; *P5CR* pyrroline-5-carboxylate reductase [EC:1.5.1.2]; *CPS1* carbamoyl-phosphate synthase (ammonia) [EC:6.3.4.16]; AOTCase N-acetylornithine carbamoyltransferase [EC:2.1.3.9]; *OTC* ornithine carbamoyltransferase [EC:2.1.3.3]; *AS* argininosuccinate synthase [EC:6.3.4.5]; *ASL* argininosuccinate lyase [EC:4.3.2.1]; *ARG* arginase [EC:3.5.3.1].

**Figure 5:**
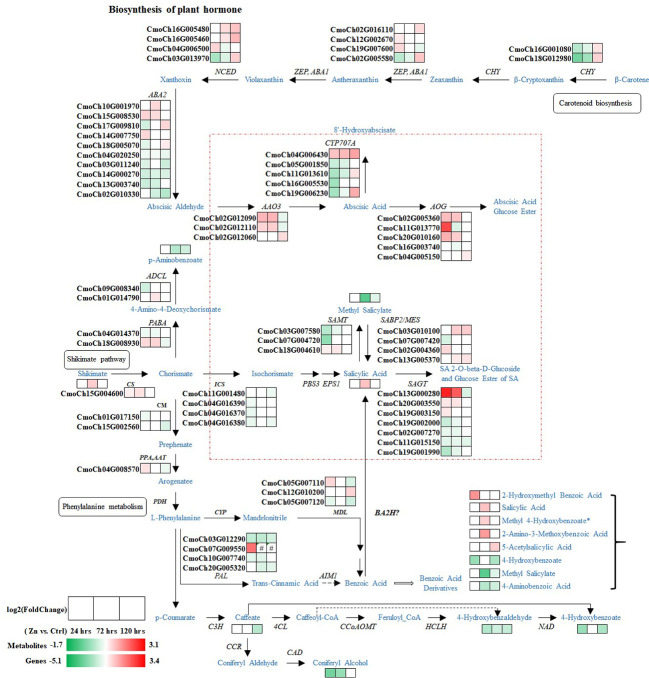
Possible biosynthesis routes for the biosynthesis of ABA and SA in the pumpkin. Blue and black colours are metabolites and genes involved in the pathway, respectively. The regulations of metabolites and genes by Zn treatments at different time-points compared to the control were demonstrated by the heatmap. Red and green colours represent the increase and decrease in abundance of metabolites or genes. The red box displays the potential core network in which relevant molecules were regulated by Zn and hence resulted in changes of ABA and SA biosynthesis in pumpkin leaves. Enzymes are abbreviated as follows: CHY carotene 3-hydroxylase [EC:1.14.15.24]; ABA1 zeaxanthin epoxidase [EC:1.14.15.21]; NCED 9-cis-epoxycarotenoid dioxygenase [EC:1.13.11.51]; ABA2 xanthoxin dehydrogenase [EC:1.1.1.288]; AAO3 abscisic aldehyde oxidase [EC:1.2.3.14]; AOG abscisate beta-glucosyl-transferase [EC:2.4.1.263]; CYP707A (+)-abscisic acid 8'-hydroxylase [EC:1.14.14.137]; ADCL 4-amino-4-deoxychorismate lyase [EC:4.1.3.38]; PABA para-aminobenzoate syn-thetase [EC:2.6.1.85]; CS chorismate synthase [EC:4.2.3.5]; ICS isochorismate synthase [EC:5.4.4.2]; PBS3 avrPphB susceptible3; EPS1 enhanced pseudomonas suscepti-bility 1; SAMT salicylic acid methyl transferase [EC: 2.1.1.274]; SABP2 salicylic acid-binding protein 2 [EC:4.1.2.10, 4.1.2.47]; SAGT SA-glucosyltransferase [EC:2.4.1.-]; CM chorismate mutase [EC:5.4.99.5]; PPA bifunctional aspartate aminotransferase and glutamate/aspartate-prephenate aminotransferase [EC:2.6.1.1, 2.6.1.78, 2.6.1.79]; PDH prephenate dehydratase [EC:4.2.1.51]; CYP cytochrome P450 [EC:1.14.-.-]; MDL mandelonitrile lyase [EC:4.1.2.10]; BA2H benzoic acid 2-hydroxylase [EC1.14.13.-]. PAL phenylalanine ammonia lyase [EC:4.3.1.24]; A1M1 abnormal inflorescence meristem1; C3H p-coumarate 3-hydroxylase [EC:1.14.13.-]; 4CL 4-coumarate:CoA ligase [EC:6.2.1.12]; CCoAOMT caffeoyl-CoA O-methyltransferase [EC:2.1.1.104]; HCLH hydroxycinnamoyl-CoA hydratase-lyase; NAD benzaldehyde dehydrogenase (NAD) [EC:1.2.1.28]; CCR cinnamoyl CoA reductase [EC:1.2.1.44]; CAD cinnamyl alcohol dehydrogenase [EC:1.1.1.195].

Followingly, to achieve a more in-depth understanding of molecular mechanisms underlying 2-AP production and accumulation in pumpkin with taro-like flavour, further analysis was applied to integrate associated pathways, including biosynthesis of amino acid, arginine biosynthesis, TCA cycle, plant hormone signal transduction, phenylalanine metabolism, and other related pathways with direct or indirect interactions (**Figures 4**, **5**).

### 3.4 Pivotal regulated genes and metabolites involved in the production of 2-AP

As mentioned above, the production of 2-AP involves several pathways, including biosynthesis of amino acids, TCA cycle, biosynthesis of arginine, and biosynthesis of arginine and proline, indicating the complex regulatory networks that involved during the process of 2-AP biosynthesis in the foliar Zn primed pumpkin. Those pathways were integrated and carefully organised to illustrate the process of 2-AP formation, and visualise the dynamic changes of associate intermediate metabolites and genes after different time intervals (**Figure 4**). The map comprehensively demonstrated the 2-AP formation related genes and metabolites, and revealed Zn-induced gene-to-metabolite changes. Zn significantly changed the abundance of 2-AP by regulating the associated genes. There were 11 metabolites with increased abundance at various time-points after Zn induction involved in 2-AP formation, including glutamate, N-acetyl ornithine, ornithine, and proline. In addition, 2-AP biosynthesis related vital genes, such as ornithine–oxo-acid transaminase (*OAT*), pyrroline-5-carboxylate reductase (*P5CR*), and delta-1-pyrroline-5-carboxylate synthetase (*P5CS*), were up-regulated, which results basically explained the increased abundance of 2-AP or related metabolites.

### 3.5 Plant hormone biosynthesis related molecular changes

Exogenous applications of stimulates can trigger the activation of downstream pathways *via* phytohormones homeostasis and their signaling networks. Phytohormone regulation initiates the biosynthesis of associated metabolites, including the volatile metabolites 2-AP and its precursor proline, by modulating genes involved in the corresponding biosynthetic pathway, thereby, affecting crop performance (Jogawat et al., 2021). As roughly illustrated in **Figure 5**, Zn activated pathways related to the synthesis of plant hormones, including salicylic acid (SA) and abscisic acid (ABA). Our study found three SA synthesis-related pathways were impacted by Zn, including the well-known isochorismate synthase (ICS) and phenylalanine ammonia lyase (PAL) pathways in plants which both start from the chorismate. Strikingly, SA accumulated at 72 hrs after the pumpkin was sprayed with Zn, but all copies of *ICS* genes were down-regulated. In addition, all three regulated *ICSs* at 24 hrs were continuously and closely distributed on the same chromosome 4 (**Figure S1**). Besides, most copies of *PAL* genes were identified with decreased expression. Only one *PAL* (*CmoCh07G009550*) was up-regulated with more than three-fold changes at the initial stage (24 hrs), while the other three genes (*CmoCh10G007740*, *CmoCh20G005320* and *CmoCh03G012290*) were all down-regulated, representing the incompatible and compatible responses to Zn treatment. The third proposed pathway in SA synthesis is associated with mandelonitrile. Mandelonitrile is another SA precursor and derived from L-phenylalanine through cytochrome P450 (CYP) and mandelonitrile lyase (MDL) (Bernal-Vicente et al., 2018). Either down- or up-regulation of three *MDLs* was identified. The last step that catalyzes benzoic acid (BA) to SA is a presumed enzyme benzoic acid hydroxylase (BA2H) (Lefevere et al., 2020), but was not identified in our study. Interestingly, the regulation of methyl salicylate (MeSA) at 72 hrs was opposite to that of SA. Salicylic acid methyl transferase (SAMT) catalyzes the methylation of SA to form MeSA (Zubieta et al., 2003). The expression of two *SAMT* genes (*CmoCh03G007580* and *CmoCh07G004720*) decreased in this study at 24 hrs and 72 hrs, which result was consistent with the down-regulated MeSA. Another vital gene related to SA biosynthesis was salicylic acid-binding protein 2 (*SABP2*) (Vlot et al., 2008). The methyl salicylate esterase activity of SABP2 can release the active defense phytohormone SA from MeSA when active SA was required. Four *SABP2* genes were identified with differential expression in the Zn treated pumpkin. In this study, three *SABP2* displayed up-regulation, among which *CmoCh02G004360* increased at the early stage (24 hrs) after Zn application. Hence, it is supposed that the increased conversion from MeSA to SA may mostly contribute to the increased concentration of SA. Another mechanism responsible for the SA accumulation may be the decreased conversion of SA to SA 2-O-beta-D-glucoside and the glucose ester of SA (SGE) by SA-glucosyltransferase (SAGT). SAGT is the key factor that negatively regulates SA levels (Kobayashi et al., 2020). There were seven *SAGT* genes regulated by Zn. Although there were three *SAGTs* up-regulated at 24 hrs, the expression abundance of those genes was generally much lower than that of the up-regulated genes. It was assumpted that down-regulated *SAGT* may play a more predominant role in regulating the SA accumulation. Except for SA and MeSA, Zn treatment induced the accumulation of another tolerance related metabolite acetyl salicylic acid (ASA) at 120 hrs, which may be the stress tolerance mechanisms induced at the later stage.

ABA is synthesized in plants through the carotenoid pathway in which the conversion of violaxanthin to xanthoxin by 9-cis-epoxycarotenoid dioxygenase (NCED) is a rate-limiting step (Chen et al., 2020). Followingly, xanthoxin dehydrogenase (ABA2) and abscisic aldehyde oxidase (AAO3) convert xanthoxin into ABA. Four *NCED*, 10 *ABA2*, and three *AAO3* genes were found with differential expression after Zn treatment. The high number of regulated ABA2 implied there may be functional redundancy. Two of three *AAO3* were up-regulated at both 24 hrs and 72 hrs, whereas the third *AAO3* gene had increased expression only at 120 hrs. Interestingly, the three *AAO3* genes were closely distributed on the same chromosome 3 (**Figure S1**). In addition, changes in ABA catabolism were discovered. Abscisic acid 8′-hydroxylase (CYP707A) catalyzes ABA to produce 8’-hydroxyabscisate (Endo et al., 2011). Five annotated *CYP707As* were identified as differentially expressed. Four of them were down-regulated at 24 hrs, while only one (*CmoCh04G006430*) was up-regulated at all time-points when compared to the control condition. Additionally, it was notable that five abscisate beta-glucosyltransferase (AOG) coded genes were regulated by Zn. In the initial of Zn treatment, three of them were regulated, and all with increased expression levels. AOG catalyzes the active ABA forming the inactive ABA conjugate ABA-glucose ester (ABA-GE), which, therefore, controls the ABA concentration and associated hormone signal in plants (Xiong et al., 2014). Although the accumulation of ABA-GE and ABA was not identified, our results indicated there may exist a tendency of increasing ABA-GE levels by promoting the generation of ABA.

### 3.6 Transcription factors

**Figure 6:**
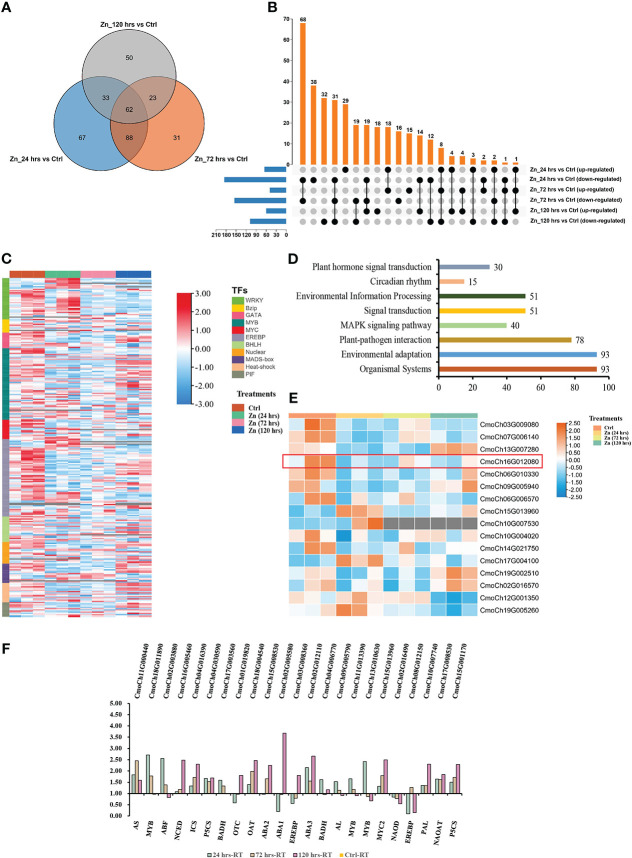
Regulated TFs in pumpkin by Zn **(A–D)**. **(A)** Venn diagram indicating the overlaps between the differentially expressed TFs in the pumpkin at 24 hrs, 72 hrs, and 120 hrs after Zn treatment, respectively. **(B)** UpSet visualisation of the number of up-regulated or down-regulated TFs in the pumpkin at three different conditions compared to the control. Filled circles and vertical lines represent the corresponding TFs (either up-regulated or down-regulated) being compared. The left bar graph for the UpSet plot shows the number of significantly increased or decreased TFs in each of the six comparisons upon Zn treatment. **(C)** Heatmap generated using the TFs with changed abundance. Red and blue colours separately indicate the relative high or low abundance in four experimental conditions. The column and row banners represent the classifications of different treatments and component classes, respectively. **(D)** Regulated TFs classified into plant hormone signal transduction and MAPK signalling pathway. **(E)** Heatmap generated with the regulated *MYC2* TFs. Orange and blue colours separately indicate the relatively high or low abundance in four experimental conditions. The column banner represents classifications of different treatments. **(F)** The expression analysis of differentially expressed genes using qRT-PCR. The x and y axises separately represent gene names and fold changes of regulated genes.

Responses of TFs to Zn were discovered in this research. A total of 354 TFs were identified with changed expression levels. In detail, there were 250 (65 up-regulated, 185 down-regulated), 204 (49 up-regulated, 155 down-regulated), and 168 TFs regulated (60 up-regulated, 108 down-regulated) at 24 hrs, 72 hrs, and 120 hrs, respectively (**Figures 6A, B**). Most TFs were down-regulated in the initial (24 hrs) as a primeval response of pumpkin to the stimulates Zn. However, the regulated TFs had the trend to recover at 120 hrs (**Figure 6C**). *MYB*, *EREBP* and *WRKY* were the three biggest TFs groups that were regulated in Zn-treated pumpkin. KEGG enrichment analysis identified significantly enriched pathways (p-value < 0.05). Interestingly, there were separately 30 and 40 regulated TFs classified into plant hormone signal transduction and MAPK signalling pathway, indicating the involvement of these TFs in signalling after Zn induction (**Figure 6D**). Overlap analysis found 19 regulated TFs were involved in two mentioned pathways, including three ethylene-responsive transcription factor 1 (ERF), and 16 *MYC2* (**Table S7**). The TFs specially involved in plant hormone signal transduction and MAPK signalling pathway were phytochrome-interacting factor (*PIF*, 11 genes), and *WRKY* TFs (21 genes), respectively. The overlapped 16 regulated *MYC2* were further analyzed. Different regulations of various *MYC2* were identified (**Figure 6E**). MEGA sequence alignment and phylogenetic tree analysis (**Figure S2A**) demonstrated that six regulated *MYC2* in pumpkin could be homologs of *MYC2* (*AT1G32640*) in *Arabidopsis thaliana* which has been proven to involve in the negative regulation of the rate-limiting enzyme P5CS1 in the biosynthesis of proline under biotic stress (Verma et al., 2019). Expression analysis indicated that *CmoCh16G012080* was the only one with both relatively high expression, and significantly differential change after Zn induction. It was down-regulated at all three time-points, which was opposite to the regulation of *P5CS1*. NCBI CD-search prediction revealed that *CmoCh16G012080* had two domains of *AtMYC2* (**Figure S2B**). The prediction of protein localization sites using PSORT (https://psort.hgc.jp/form.html) elucidated that the gene was located in the nucleus of the cell, which further demonstrated the potential activities of this MYC2 on the nuclear DNA in pumpkin. The homologous sequence alignment was further confirmed using the online tool Clustal Omega (https://www.ebi.ac.uk/Tools/msa/clustalo/) (**Figure S2C**). *CmoCh16G012080* and *AtMYC2* share 58.28% of identity, indicating *CmoCh16G012080* could be the potential MYC2 regulating the proline biosynthesis in pumpkins. Besides, to validate the transcriptome data, the relative expression levels of research associated genes were analyzed using qRT-PCR, and the results were proved to be reliable (**Figure 6F**).

## 4 Discussion

### 4.1 Zn treatments induced the gene-to-metabolite regulation in pumpkin

The integration analysis of metabolome and transcriptome comprehensively illustrated the Zn induced gene-to-metabolite regulation in pumpkin leaves. In addition, a time-dependent manner of molecule changes was observed in pumpkin after Zn priming and foliar application. Delightfully, the metabolic analysis found the 2-AP was significantly accumulated, indicating the successful induction of this compound by foliar Zn treatment, and the potential of Zn application in improving the taro-like aroma associated quality characters of pumpkin leaves. The integrated map using metabolites and genes comprehensively and firstly visualized the main pathways involved in the 2-AP formation and its correlative regulation in pumpkin leaves after Zn stimulation, and changes in abundance of related molecules, underlying there were complex regulations during the process of 2-AP production and aroma formation.

### 4.2 Proline and ornithine generation contributed to the accumulation of 2-AP

Illustrating the molecular mechanism of 2-AP production is important in improving the aroma quality of pumpkin leaves. 2-AP biosynthesis involves in important precursors or intermediates, and key enzymes (Bao et al., 2021). Proline is the most important precursor of 2-AP. This protein derived amino acid has been reported with multi-functions, and well-acknowledged to play an important role as an osmolyte which helps stabilize protein structure and contributes to ROS scavenging (Szabados and Savoure, 2010). This point was clarified in young plant species *Cucurbita pepo* L. and *Cucurbita moschata* Poir under salt stress. Antioxidant enzymes activities increased in the salt and proline treated plants very effectively compared to control plants under salt stress (Yoshihashi et al., 2002; Szabados and Savoure, 2010). Six superoxide dismutase (*SOD*) genes were identified as differentially expressed at different time intervals after the Zn application. Four of them were significantly up-regulated at 72 hrs, which was coincidence with the increased proline at 72 hrs.

It was reported that higher amount of proline promoted the 2-AP accumulation and improved the aroma profile of the fragrant rice (Yoshihashi et al., 2002; Bao et al., 2021). Additionally, increased 2-AP occurred in rice seedlings and callus when the intermediates or precursors were supplied, such as proline, ornithine, and glutamate. In particular, the proline increased the 2-AP concentration by more than threefold (Yoshihashi et al., 2002). Our experimental findings were consistent with these previous reports, as both increased proline and 2-AP were detected in pumpkin leaves. Thus, proline biosynthesis is vital for the 2-AP formation. Proline is synthesized predominantly from glutamate, in which enzymes P5CR and P5CS are of the utmost importance during this process (Liang et al., 2013). Both *P5CR* and *P5CS* genes had increased expression after the Zn treatment in this study. It is remarkable that ornithine was the alternative source for proline biosynthesis (Winter et al., 2015). The increased ornithine was found at both 24 hrs and 72 hrs. The yield of ornithine also starts from glutamate, followed by the acetylation, phosphorylation, reduction, and transamination (Winter et al., 2015). Vital enzymes in the pathway, including *NAGS, NAGK, APGR*, and *ACOAT* were identified, and had increased expressions, indicating those genes may regulate the formation of the downstream ornithine, citrulline, and arginine by regulating their expression patterns. Therefore, the proposed molecular mechanism related to 2-AP formation in pumpkin leaves in our study is that Zn induced the increase of 2-AP associated precursors ornithine and proline, which contribute to the 2-AP accumulation. Key genes (*P5CS*, *P5CR*, *NAGS*, *NAGK*, *APGR*, *ACOAT*, and *OAT*) were mostly up-regulated in pumpkin leaves by Zn, and therefore, positively catelyse the production of associated 2-AP precursors or intermediates. As previously described, 2-AP formation may occur in both leaves and grains of rice (Hinge et al., 2016; Pan et al., 2021). In addition, previous studies identified this component in pumpkin leaves, fruits and seeds (Poehlmann and Schieberle, 2013; Li et al., 2019; Li et al., 2022). Potentially, findings in this study can help the better understanding of 2-AP accumulation in other tissues by keeping track of the expression of key genes and the production of precursors associated with 2-AP content, together with the synthesis and translocation of aroma volatiles across the various developmental stages.

### 4.3 Changes in primary metabolism played an important role during the process of aromatic compounds production

Zn, as an important micronutrient, is essential to the function of many enzymes in carbon and nitrogen metabolism, energy transfer and protein synthesis (Sung, 2020). Our study indicated that carbon metabolism was one of the most significantly enriched pathways at three time-points after the foliar Zn application. Carbon metabolism belongs to the primary metabolism, and has tight connection between the carbon metabolism and the aroma formation. Many primary metabolites that generated from primary metabolism are the direct precursors of aromatic compounds. Actually, aroma profile is the combination of hundreds of volatiles which can be generated from the primary metabolites (Pott et al., 2019).

In addition, amino acid associated pathways were also enriched by KEGG pathway analysis. Amino acid biosynthesis and metabolism are tightly connected with other biological pathways, including energy and carbohydrate metabolism, protein synthesis, carbon-nitrogen budget, stress responses, and hormone and secondary metabolism. There were 83 amino acids identified from pumpkin at 72 hrs after Zn treatment, with 14 of them being regulated (eight and six amino acids were separately down- and up-regulated). Increased levels of proline, citrulline, N-α-acetyl-L-ornithine, ornithine and arginine were particularly identified at 72 hrs, as mentioned above. Nitrogen-metabolizing pathways in plants generate ornithine, arginine, proline and polyamines through glutamate metabolism. During this process, plants produce intermediates which are vital to plant development and responses to various environmental changes (Majumdar et al., 2016). Understanding the key enzymatic reactions important for the production of primary precursors can hopefully help the transfer of metabolic flux from the central to the specialized metabolism processes, which potentially increases the formation of the final products generated from the specified biosynthetic pathway (Pott et al., 2019). Zn priming and foliar application mimicked the environmental changes. The connection between primary metabolism and aroma formation, and associated regulation are key factors for breeding crop cultivars with enhanced aroma.

### 4.4 Plant hormones involved in the regulation of 2-AP biosynthesis

Plant hormones are important signal molecules in sorts of signaling pathways as these pathways are directly or indirectly correlated in a wide of abiotic and biotic stress responses (Jogawat et al., 2021). The exogenous Zn, acted as the regulatory element, involves in the biosynthesis of plant hormones, and therefore, impact gene expression, could be a controversial yet interesting research direction. Zn-induced changes of genes and metabolites involved in the SA and ABA biosynthesis may affect the 2-AP formation by regulating the expression of key genes controlling the biosynthesis of specific compounds. In this study, phytohormone, as the messenger, may be involved in signal transduction systems, and induced the particular enzymes encoded genes in the proline metabolic pathway, therefore, produced associated defense compounds, including proline itself.

Kobra Maghsoudi et al. found SA elevated the expression of *P5CS*, and increased formation of proline in wheat under drought stress (Maghsoudi et al., 2018). Significantly increased SA and proline were exactly discovered at 72 hrs after Zn treatment in this study, indicating the potentially direct or indirect regulation between those two biomolecules. Three SA biosynthesis pathways were induced by Zn, but had potential different performance on SA formation. It is notable that the well-known ICS pathway in which *ICS* encodes key enzymes for SA production was negatively regulated after Zn application. All regulated *ICSs* at 24 hrs were decreased, and continuously and closely distributed on the same chromosome 4 of pumpkin, which was not identified in other species. The close distribution of genes with the same function may represent the function redundency, or the enhanced negative regulation on ICS pathway. As reported, the predominance of different pathways related to SA biosynthesis can be different within the plants (Lefevere et al., 2020). Complex coordination among different pathways may be involved in the regulation of SA biosynthesis in pumpkin after Zn treatment in this study.

Correlation between ABA and proline was found in rice under hypoxic stress (Cao et al., 2020). ABA is believed to regulate genes relevant to proline biosynthesis, and act on upstream of the proline formation, which enhances the rice resistance and alleviates the impact of stress (Cao et al., 2020). ABA accumulated in drought-stressed plants seems to be involved in the proline synthesis by regulating the key gene *P5CS* at the transcriptional level (Bandurska et al., 2017). Although ABA accumulation was not found in this study, the associated mechanism was activated. For example, gene *AAO3* that involves in the last step of ABA biosynthesis, was discovered and up-regulated in this study. There is evidence that this gene positively regulates ABA level, and is essential for seed germination, seedling growth, grain yield, and drought tolerance in rice (Shi et al., 2021). Potentially, the biosynthesis of SA and ABA in plants can be customized so that the metabolite assembly can be redirected to specific needs.

### 4.5 Transcription factors acted as regulators of aromatic amino acid biosynthesis

Transcription factors involve in numerous life processes by either directly or indirectly regulating the downstream target genes. WD40, MYB, WRKY, bHLH, and bZIP families are the most common TFs among the over 60 TF families found in higher plants. These TFs were also discovered with differential expression in pumpkin leaves after Zn treatment in this study (Song et al., 2021). As described, MYB, EREBP and WRKY were the Zn-induced three largest regulated TFs groups. This finding was consistent with the results demonstrated in foliar Zn applied rice leaves where MYB and WRKY were the major differentially expressed TF families after the treatment and may play a crucial role during 2-AP biosynthesis (Imran et al., 2022). Study found MYB TFs regulate the biosynthesis of aromatic amino acids, and the downstream secondary metabolites in rice, including the proline (Liu et al., 2015). WRKY TFs were involved in the regulation of *PAL* and *ICS* genes, which was correlated with the SA accumulation in rice (Choi et al., 2015). Notably, it was reported that *AtMYC2* in *Arabidopsis thaliana* can act as the regulatory hub within several signaling pathways. And the new role of *AtMYC2* was to regulate the *P5CS1* gene and hence proline biosynthesis (Verma et al., 2019). In this study, massive regulated *MYC2* were involved in both plant hormone signal transduction and MAPK signalling pathway. Potentially, homologs of *AtMYC2* in Zn treated pumpkin and their differential expression may be functional in the regulation of phytohormone signaling and their target genes (such as *P5CS*), therefore, regulate the proline and 2-AP synthesis. Additionally, other differentially expressed TFs in Zn treated pumpkin leaves, such as MADS-box TFs, heat shock TFs, ethylene-responsive TFs, and bHLH TFs, are also reported to be involved in the corresponding regulatory mechanisms that integrate phytohormone and redox signaling in the plant response to a number of stresses, therefore, directly or indirectly impact the 2-AP biosynthesis (Trivedi et al., 2016). Further studies are required to better understand the role of specific TFs in 2-AP biosynthesis in pumpkin leaves, especially those that directly impact the associated key genes in the pathway.

### 4.6 Zn induced other metabolites changes, together with 2-AP, contributed to the overall aroma profile of pumpkin

Except for increased 2-AP, the cis-3-hexen-1-ol was detected by GC-MS, but significantly down-regulated at all three time internals compared to the control. The cis-3-hexen-1-ol, as the most influential component of green aroma from plants, is a valuable resource for imparting fruit and vegetable aromas. It was reported that the hyperosmotic stress can induce the accumulation of cis-3-hexen-1-ol and up-regulation of most associated genes (such as *AHDs*) in *Camellia sinensis*, while in turn, this component could considerably improve the hyperosmotic stress tolerance through reducing the stomatal conductance and MDA, increasing ABA and proline generation (Hu et al., 2020), which pattern of regulation was different with our study. The cis-3-hexen-1-ol can be generated from 3-hexenal by the catalyzation of alcohol dehydrogenase (ADH, EC 1.1.1.1) through the alpha-linolenic acid metabolism (Jin et al., 2016). Transcriptome comparative analysis identified three *AHDs* (*CmoCh02G009540*, *CmoCh05G010180* and *CmoCh05G010190*) with decreased expression, which may result in the decreased production of cis-2-hexen-1-ol. The cis-3-hexen-1-ol with green aroma, geraniol with rose-like fragrance, 2-AP with popcorn flavour, together with other regulated aromatic compounds, contributed to the aroma profile in pumpkin leaves after Zn priming and foliar application.

## 5 Conclusions

Aroma of the fresh pumpkin leaves can affect the perception of consumers, and therefore the determination of their preference. The interplay of theoretical and practical have summarized that the character improvement of crops can be achieved by artificially changing the environmental conditions surrounding the plants appropriately. It is manifested that foliar application of Zn was efficient in improving the 2-AP formation in pumpkin leaves. We hypothesis that Zn modulates the 2-AP biosynthesis in a complex way at transcriptional level. The up-regulated *P5CS* after Zn treatment was supposed to contribute to the accumulated proline, and as a result, promtoed the 2-AP accumulation. It is concluded that *P5CS* is Zn-inducible, which makes it a potential gene for aroma quality improvement of pumpkin leaves. In addition, Zn may directly or indirectly interacts with plant hormones. ABA and SA, together with the transcription factors (such as MYB and MYC), were deemed to play the critical role in the elevation of 2-AP production in the metabolite profile. It is supposed that Zn application regulated the primary metabolism, which contributed to the change of aromatic components. Phytohormones may interact with Zn, and act as the signal molecule and regulate the aroma formation relevant biological processes. TFs that control specific pathways appear to be the promising tool for metabolic engineering with the aim to improve the aroma profile of pumpkins.

## Data availability statement

The datasets presented in this study can be found in online repositories. The names of the repository/repositories and accession number(s) can be found below: CNGB Sequence Archive (CNSA) of China National Genebank DataBase, CNP0003866.

## Author contributions

JxL and XY designed and planned the experiments. JxL performed the GC-MS experiment. YQ, XY, JnL, HG, HW and XZ prepared the samples. LD, XL, GZ and JxL analyzed the data. LD and JxL wrote and reviewed the article. All authors contributed to the article and approved the submitted version.

## Supplementary material

Supplementary Figure 1 The Circos distribution of differentially expressed genes on chromosomes associated with the biosynthesis of 2-AP and hormones from and . The red and green colors separately represent the up and down regulation at 24 hrs **(A)**, 72 hrs **(B)** and 120 hrs **(C)** after Zn treatment. The size of red and green filled circles indicates the fpkm value of corresponding up- and down-regulated genes (the name on the map consists of 2AP (2-AP biosynthesis related) or Hor (hormone biosynthesis related), gene ID, and gene name), the bigger size represents higher expression level.

Supplementary Figure 2 Regulated MYC2 TFs in Zn treated pumpkin leaves. **(A)** MEGA sequence alignment and phylogenetic tree analysis using the regulated MYC2 TFs and AtMYC2. **(B)** NCBI CD-search prediction of CmoCh16G012080 and AtMYC2. **(C)** Homologous sequence alignment of CmoCh16G012080 and AtMYC2 using the online tool Clustal Omega.
