# Supplementary figures and images for: Metabolic and molecular mechanisms underlying the foliar Zn application induced increase of 2-acetyl-1-pyrroline conferring the ‘taro-like’ aroma in pumpkin leaves
Source: Front Plant Sci. 2023 Jan 26;14:1127032. doi: 10.3389/fpls.2023.1127032 (PMC9909474; doi:10.3389/fpls.2023.1127032)

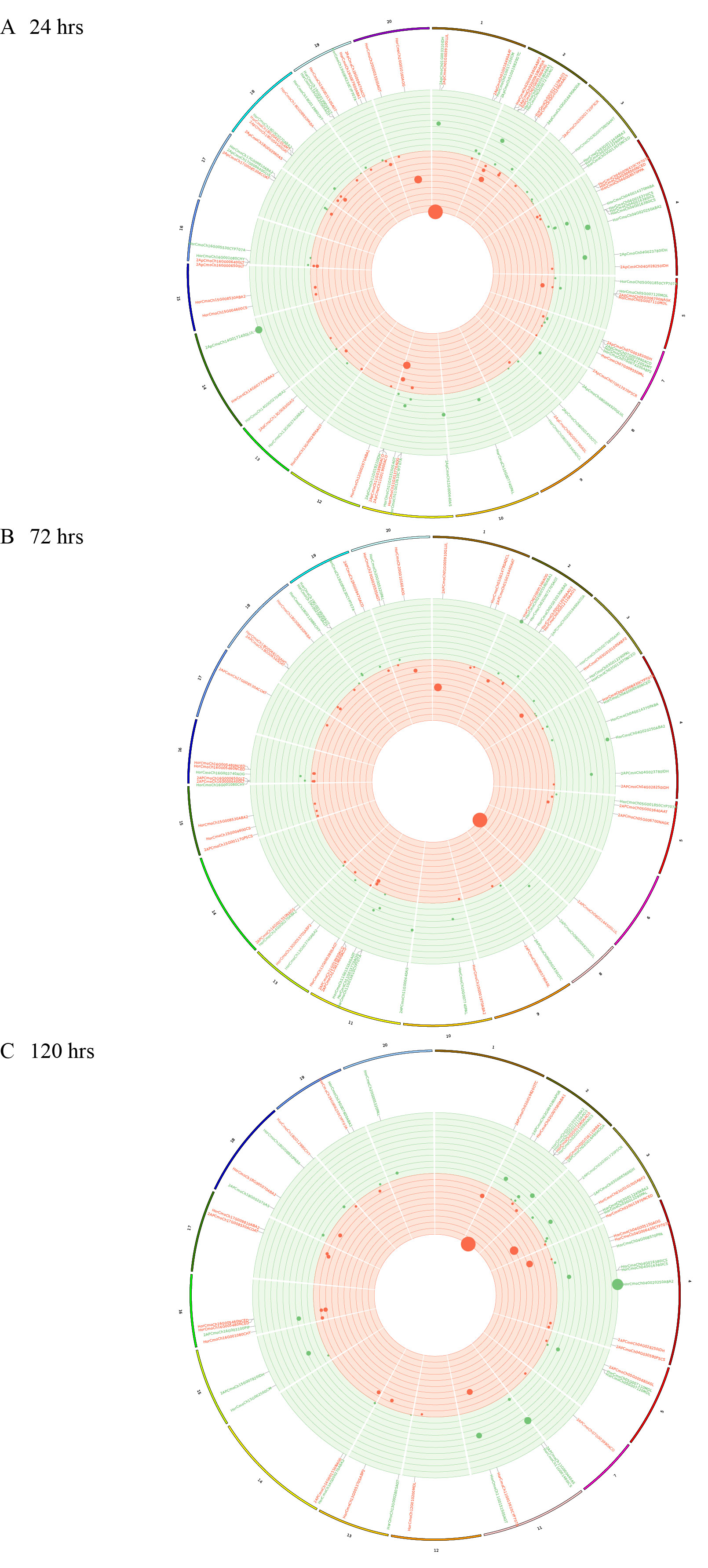

Supplement: Supplementary Figure 1 — The Circos distribution of differentially expressed genes on chromosomes associated with the biosynthesis of 2-AP and hormones from and . The red and green colors separately represent the up and down regulation at 24 hrs (A), 72 hrs (B) and 120 hrs (C) after Zn treatment. The size of red and green filled circles indicates the fpkm value of corresponding up- and down-regulated genes (the name on the map consists of 2AP (2-AP biosynthesis related) or Hor (hormone biosynthesis related), gene ID, and gene name), the bigger size represents higher expression level. [file Image_1.jpeg]

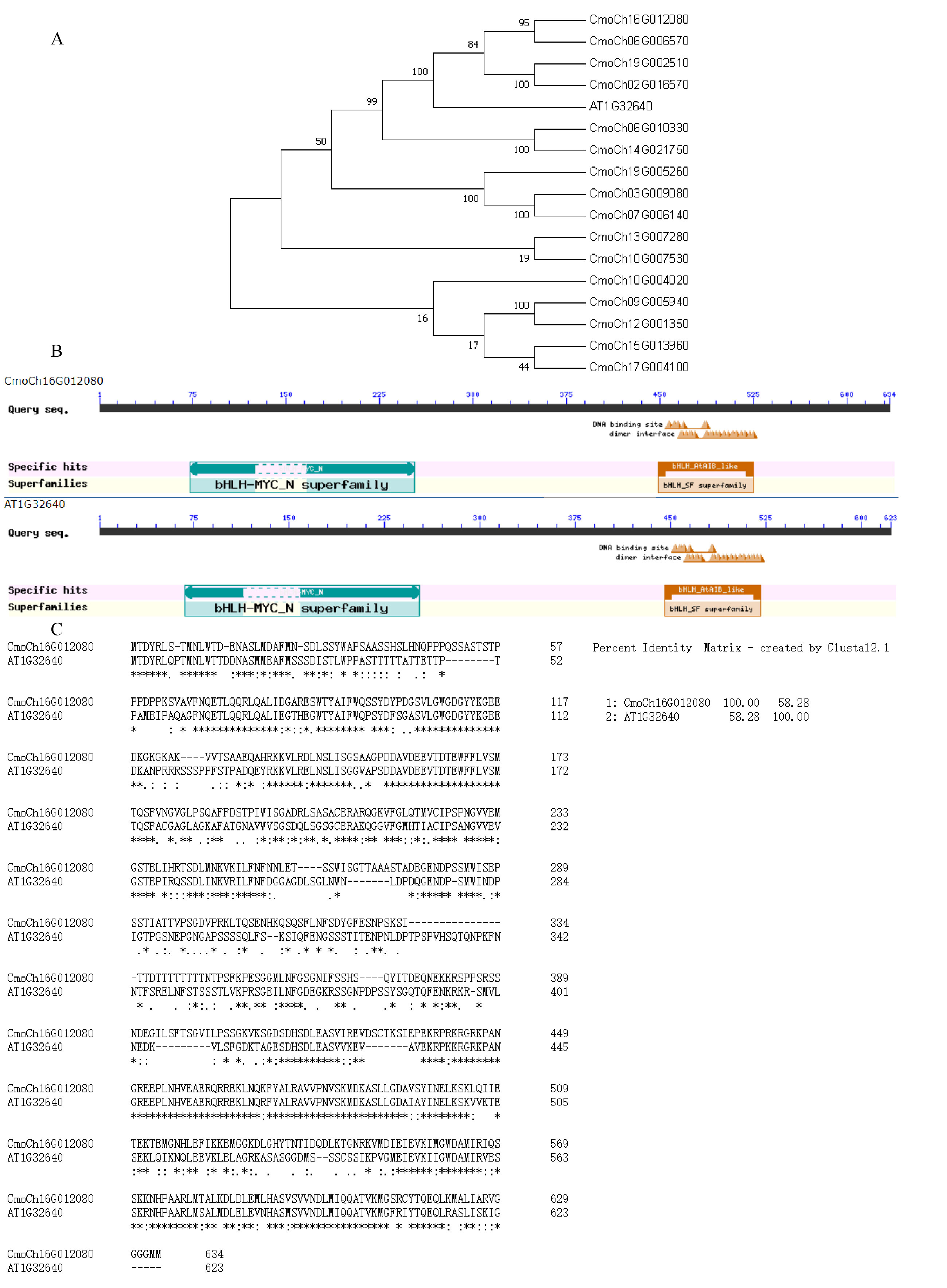

Supplement: Supplementary Figure 2 — Regulated MYC2 TFs in Zn treated pumpkin leaves. (A) MEGA sequence alignment and phylogenetic tree analysis using the regulated MYC2 TFs and AtMYC2. (B) NCBI CD-search prediction of CmoCh16G012080 and AtMYC2. (C) Homologous sequence alignment of CmoCh16G012080 and AtMYC2 using the online tool Clustal Omega. [file Image_2.jpeg]
